# Resveratrol and Alzheimer’s disease: message in a bottle on red wine and cognition

**DOI:** 10.3389/fnagi.2014.00095

**Published:** 2014-05-14

**Authors:** Alberto Granzotto, Paolo Zatta

**Affiliations:** ^1^Molecular Neurology Unit, Center of Excellence on Aging (Ce.S.I.)Chieti, Italy; ^2^CNR-Institute for Biomedical Technologies, Padua “Metalloproteins” Unit, Department of Biology, University of PaduaPadua, Italy

**Keywords:** resveratrol, Alzheimer’s disease, aging, metal ions, aluminum, copper, iron, zinc

## Abstract

Cognitive impairment is the final outcome of a complex network of molecular mechanisms ultimately leading to dementia. Despite major efforts aimed at unraveling the molecular determinants of dementia of Alzheimer type (DAT), effective disease-modifying approaches are still missing. An interesting and still largely unexplored avenue is offered by nutraceutical intervention. For instance, robust epidemiological data have suggested that moderate intake of red wine may protect against several age-related pathological conditions (i.e., cardiovascular diseases, diabetes, and cancer) as well as DAT-related cognitive decline. Wine is highly enriched in many polyphenols, including resveratrol. Resveratrol is a well recognized antioxidant which may modulate metal ion deregulation outcomes as well as main features of the Alzheimer’s disease (AD) brain. The review will discuss the potentiality of resveratrol as a neuroprotectant in dementia in relation to the oxidative stress produced by amyloid and metal dysmetabolism.

## Introduction

The so-called *French paradox* arises from the epidemiological fact that French people, despite their indulgence to a high fat diet, show a relative low incidence of cardiovascular diseases (Renaud and De Lorgeril, 1992). Several epidemiological studies have shown that moderate wine consumption can be effective in slowing down age-related cognitive decline (Wang et al., 2006; Panza et al., 2012; Corona et al., 2013). A possible explanation of this phenomenon has been linked to the national high consumption of wine (20–30 g/day) (Renaud and De Lorgeril, 1992). Albeit moderate ethanol intake is, generally speaking, “beneficial”, some more specific effects appear to be related to red wine. Red wine consumption seems in fact to promote far more protective effects than consumption of other ethanol containing beverages (Baur and Sinclair, 2006). Resveratrol, a natural polyphenol, is mainly present in red wine and has been suspected to be the major driving force behind the *French paradox* (Siemann and Creasy, 1992).

AD is one of the most common forms of dementia in the elderly. To date, no disease-modifying therapies are still available for AD.

The main four pathological features of the disease are: (1) extracellular deposition of misfolded β-amyloid (Aβ) in senile plaques (SPs); (2) intracellular accumulation of hyperphosphorylated tau in neurofibrillary tangles (NFTs); (3) severe brain atrophy; and (4) the presence of areas of chronic inflammation (Querfurth and Laferla, 2010; Medeiros et al., 2013).

In the last 20 years, deregulation of Aβ metabolism (amyloid oligomerization, aggregation, and plaques formation) has been considered the main trigger for AD-related synaptic dysfunction. Amyloid has been therefore the major target for therapeutic intervention (Hardy and Higgins, 1992; Mucke and Selkoe, 2012). Unfortunately, most of these attempts have dramatically failed or have produced only marginal effects (Reitz, 2012; Krstic and Knuesel, 2013; Doody et al., 2014).

Better therapeutic strategies are thus needed along with new acknowledgment that AD is a complex multifactorial syndrome.

Aging is the required paramount condition (Herrup, 2010) on which, in addition to Aβ together with tau deregulation, genes, chronic inflammation, mitochondrial, metabolic dysfunctions, impaired insulin signaling, oxidative stress, aberrant cell cycle reentry, cholesterol dysmetabolism as well as metal ion dyshomeostasis must synergistically work to promote AD pathological manifestation (Herrup, 2010; Querfurth and Laferla, 2010; Roberts et al., 2012). While a single-target therapeutic strategy seems to produce only suboptimal results a broader neuroprotective approach, at least theoretically, appears more appealing (Mudher and Lovestone, 2002).

In this review, we are providing some evidence for resveratrol as a broad-spectrum neuroprotective agent in aging and hopefully in AD.

## Resveratrol

Resveratrol has beneficial cardiovascular effects (Siemann and Creasy, 1992) throughout a great variety of molecular mechanisms (Howitz et al., 2003; Baur and Sinclair, 2006; Lagouge et al., 2006; Park et al., 2012).

**Figure 1:**
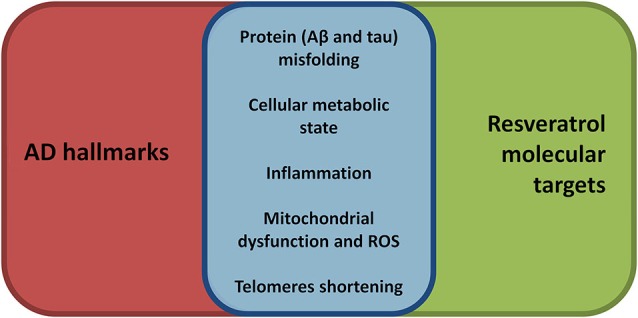
**Synoptic view of the overlap between aging, and thus AD, hallmarks (see López-Otín et al., 2013) and the molecular targets of resveratrol**. Resveratrol, compared to many other disease modifying approaches, shows a broader range of beneficial effects targeting many molecular aspects of AD pathogenesis. Thus, resveratrol may represent a promising broad-spectrum neuroprotective agent in AD.

A recent review on aging determinants has proposed nine hallmarks for the process (López-Otín et al., 2013). Not surprisingly, almost all of them are also involved in AD development and progression (Figure 1; Herrup, 2010; Querfurth and Laferla, 2010) and, notably, at least five, are well recognized target for resveratrol modulation.

In the following sections we have outlined potential effects of resveratrol on these aging and/or AD molecular targets.

### β-amyloid and hyperphosphorylated tau misfolding

Blockade of Aβ deposition into SPs and inhibition of hyperphosphorylation of tau into NFTs has been considered mandatory to prevent or, at least, delay AD-related cognitive decline.

Resveratrol has been shown to inhibit Aβ fibrils formation (Porat et al., 2006; Rivière et al., 2007). Moreover, *in vitro* and *in vivo* studies have also indicated that resveratrol reduces amyloid toxicity by decreasing Aβ production through sirtuin-dependent activation of a disintegrin and metalloproteinase domain-containing protein 10 (Donmez et al., 2010). The compound also increases clearance and metabolism via an AMP-activated protein kinase-pathway and can induce autophagic and lysosomal Aβ degradation (Marambaud et al., 2005; Vingtdeux et al., 2010). Resveratrol can effectively interject in the amyloid cascade through its antioxidant and anti-inflammatory activity, thereby reducing Aβ-driven production of reactive oxygen species (ROS) as well as neuroinflammation (Liu and Bitan, 2012).

Effects on tau phosphorylation and deposition have been less investigated. However, resveratrol-mediated activation of sirtuin-1 (SIRT1) can lead to direct deacetylation of acetylated tau, thereby promoting its proteasomal degradation (Min et al., 2010). In addition, the compound can reduce phospho-tau toxicity (induced by cyclin-dependent kinase 5-p25 dependent tau phosphorylation) by favoring the deacetylation of peroxisome proliferator-activated receptor gamma, coactivator 1 alpha (PGC-1α) and p53 (Kim et al., 2007).

### Cellular metabolism

Caloric restriction has been proposed to be effective in increasing lifespan in several animal models. Fasting has been observed to promote beneficial effects on preclinical models of AD and aging not only by extending lifespan but also by ameliorating cognitive performances (Halagappa et al., 2007). Caloric restriction can in fact promote release of brain-derived neurotrophic factor (BDNF), a neurotrophin critically involved in counteracting cognitive decline (Weinstein et al., 2014).

In this context, resveratrol efficiently mimics caloric restriction by inducing expression of SIRT1 (a nicotinamide adenine dinucleotide (NAD^+^) dependent deacetylase) which in turn sets in motion a cascade of PGC-1α-dependent events that ultimately lead to improved mitochondrial functioning and biogenesis and boost cellular ROS scavenging (Gomes et al., 2013; López-Otín et al., 2013).

### Inflammation

Areas of localized inflammation and active microglia contribute to neurodegeneration and cognitive decline in AD brains (McGeer and McGeer, 2013). Pharmacological and genetic manipulations aimed at reducing brain inflammation appear to be effective in slowing/modifying the disease progression in AD animal models (Heneka et al., 2013; Giuliani et al., 2014).

Resveratrol is effective in reducing the inflammatory status (Rahman et al., 2006; Chen et al., 2013) in *in vitro* and *in vivo* settings of neuroinflammation (Capiralla et al., 2012; Frozza et al., 2013).

Mechanisms by which resveratrol attenuate neuroinflammation are still not completely clear. A major pathway seems to involve sirtuin-dependent arrest of nuclear factor kappa-light-chain-enhancer of activated B cells signaling cascades, a step that results in downstream blockade of microglia activation (Capiralla et al., 2012; Donmez, 2012; Ye et al., 2013).

### Mitochondrial dysfunction and ROS

Mitochondria play an essential role in the cell wellbeing. The organelles critically control cellular energy and metabolism as well as intracellular signaling (Rizzuto et al., 2012). On the dark side, mitochondria are also key players in modulating cellular death through release of apoptotic factors, blockade of energy supply and generation and release of ROS. Alterations of mitochondrial functioning are known in aging and early stages of AD (Wang et al., 2013).

Mitochondrial electron leakage, followed by ROS production occurs in neurodegenerative conditions paving the way to lipid peroxidation, nucleic acid damage, protein oxidation, and, eventually, neuronal death (Wang et al., 2013).

Resveratrol counteracts the production of mitochondrial ROS through two major mechanisms: (1) by efficiently scavenging hydroxyl, superoxide, and metal-induced radicals (Leonard et al., 2003); and (2) by increasing mitochondrial functioning and biogenesis through activation of the SIRT1–PGC-1α pathway, thereby boosting mitochondrial bioenergetic efficiency (Khan et al., 2012; Choi et al., 2013; Desquiret-Dumas et al., 2013).

### Telomeres shortening

Telomeres shortening plays a key role in cellular aging and AD (Cai et al., 2013; Mathur et al., 2014). Short telomeres increase DNA vulnerability to stressful insults (i.e., UV irradiation, ROS production) ultimately leading to aberrant cell functioning and cell death. Polyphenols has a positive impact upon maintenance of telomeres length (Jayasena et al., 2013). In that respect, resveratrol promotes the expression of Werner syndrome ATP-dependent helicase, a telomere maintenance factor (Uchiumi et al., 2011), increases the activity of telomerase via a SIRT1-dependent pathway (Palacios et al., 2010), and spares telomeres and DNA from ROS dependent damages thanks to its intrinsic scavenging properties (Jayasena et al., 2013).

## Metal imbalance in the AD brain: a potent trigger of oxidative stress

In the brain, metal ions are involved in many essential processes such as intracellular signaling, modulation of cellular redox and metabolic states, enzymatic activities and channels functioning (Billard, 2006; Sensi et al., 2009; Rizzuto et al., 2012; Sekler and Silverman, 2012; Gaier et al., 2013). Metal homeostasis is strictly controlled by the interplay of transporters, channels, chaperones and metalloregulatory sensors (Finney and O’halloran, 2003). In neurodegenerative conditions and/or aging, this tightly controlled system is lost, thereby leading to disease-promoting metal imbalance (Bolognin et al., 2009; Breydo and Uversky, 2011; Jellinger, 2013).

Metal ion dyshomeostasis is in fact involved in several neurological disorders like Parkinson’s disease (PD), Amyotrophic Lateral Sclerosis (ALS), Prion Protein disease, Huntington’s disease (HD), and AD. All these neurodegenerative conditions share common pathological features that include deposition of misfolded proteins, metal ion deregulation and exposure to oxidative stress (Boillee et al., 2006; Duce and Bush, 2010; Roberts et al., 2012; Gonzalez-Dominguez et al., 2014).

In AD, metal ion dyshomeostasis represents a key, though too often overlooked, pathological step. A metal hypothesis for AD has been proposed by many authors (Bush, 2008). In that respect, copper, iron, zinc and aluminum are the metals found deregulated in AD. All of them are able to alter Aβ metabolism and deposition (Bolognin et al., 2011). SPs but also NFTs are highly enriched of these metals. Moreover, all these ions can promote ROS generation (Sayre et al., 2000; Granzotto and Zatta, 2011; Pithadia and Lim, 2012; Ayton et al., 2013).

Recent findings have shown that low levels of copper are sufficient to dramatically affect Aβ homeostasis by increasing Aβ accumulation and neuroinflammation related to Aβ-deposition (Singh et al., 2013). Compared to nondemented elderly controls, brains of AD patients show an increased presence of labile copper pools, which correlate with oxidative damage in these tissues (James et al., 2012). Resveratrol is a well known copper chelator (Tamboli et al., 2011) and, in theory, of some use in AD (Faux et al., 2010). Unfortunately, the copper-resveratrol complex seems to be more harmful than beneficial in the context of AD. Resveratrol promotes the reduction of copper (II) to copper (I) (de la Lastra and Villegas, 2007) and several studies have indicated a pro-oxidant activity of the compound when bound to copper (Zheng et al., 2006; de la Lastra and Villegas, 2007; Muqbil et al., 2012). Thus, resveratrol activity on copper homeostasis appears more harmful than neuroprotective if used as standalone therapeutic approach. A feasible and, in our opinion, clinically relevant approach might be represented by the administration of resveratrol in association with a higher affinity copper chelator. This would lead, at least in theory, to a dual beneficial effect: reduction of copper dyshomeostasis coupled with decreased ROS production.

Iron deregulation has been linked to AD (Weinreb et al., 2013; Crespo et al., 2014; Gonzalez-Dominguez et al., 2014). Role of iron in AD pathogenesis is substantiated by the effectiveness of metal homeostatic therapies aiming at reducing iron deregulation (Crouch et al., 2007), which results in (1) decreased free iron accumulation and ferroptosis (Dixon et al., 2012); (2) decreased iron-dependent ROS production; and (3) blockade of neurotoxic Aβ-iron conjugates formation (Liu et al., 2012). To date, *in vivo* evidence for iron chelation by resveratrol is missing, however the compound prevents iron-driven mitochondrial dysfunction by inhibiting glycogen synthase kinase-3 beta activity (a mechanism useful also to prevent tau hyperphosphorylation) (Shin et al., 2009), and by reducing peroxidation of lipoproteins and lipids through its activity as scavenger (Belguendouz et al., 1997; Tadolini et al., 2000).

Zinc dyshomeostasis has been proposed as a risk factor for AD. Accumulation of excessive zinc, or its deficiency, are both involved in the neuronal loss which leads to AD and aging related cognitive decline (Brewer, 2012). While zinc deficiency increases neuroinflammation and also affects BDNF maturation and ultimately cognition, aberrant intracellular zinc mobilization or accumulation leads to mitochondrial failure and ROS production. Extracellular zinc overload within SPs also inhibits the iron-export ferroxidase activity further increasing ROS production and ultimately neuronal death (Duce et al., 2010). Resveratrol does not directly affect zinc levels however it can be useful in preventing the full development of zinc-dependent injurious mechanisms. Actually, resveratrol inability to sequester zinc does not represent a limitation, as the compound can exert antioxidant activities without producing zinc deficiency.

Aluminum lacks modulatory functions in biological processes; however, its accumulation in the brain has been demonstrated to be linked to several neuropathological conditions (Zatta et al., 2003; Walton, 2013). To date three are the main mechanisms through which aluminum exerts its neurotoxic effects: (1) production of ROS; (2) induction of neuroinflammation; and (3) formation of toxic aggregates of misfolded proteins (Perl, 2006; Kumar et al., 2009; Wu et al., 2012; Bolognin et al., 2013). In AD, aluminum seems to act as an effective cross-linker between tau phospho-sites, to “freeze” Aβ in its toxic oligomeric state, and to induce exposure of Aβ hydrophobic clusters aggregates, thereby boosting toxic properties of these misfolded proteins (Zatta et al., 2009; Bolognin et al., 2011; Chen et al., 2011; Granzotto et al., 2011). Aluminum-related oxidative damage occurs through lipid peroxidation, alteration of the activity of antioxidant enzymes, alterations of mitochondrial functioning and biogenesis and promotion of DNA injury (Zatta et al., 2002; Sharma et al., 2013). Resveratrol shows a negligible ability to bind aluminum *in vitro* (Granzotto and Zatta, 2011), nevertheless, it seems effective in reducing *in vivo* the downstream events of aluminum overload, namely the aluminum-related ROS production and neuroinflammatory response activation (Zaky et al., 2013).

## Conclusions

Resveratrol is a multi target compound and may represent an effective therapeutic tool in aging-related neurodegenerative processes. Consistently, several clinical trials are ongoing to test its effectiveness as dietary supplement to slow dementia progression (ClinicalTrials.gov, 2014).

**Figure 2:**
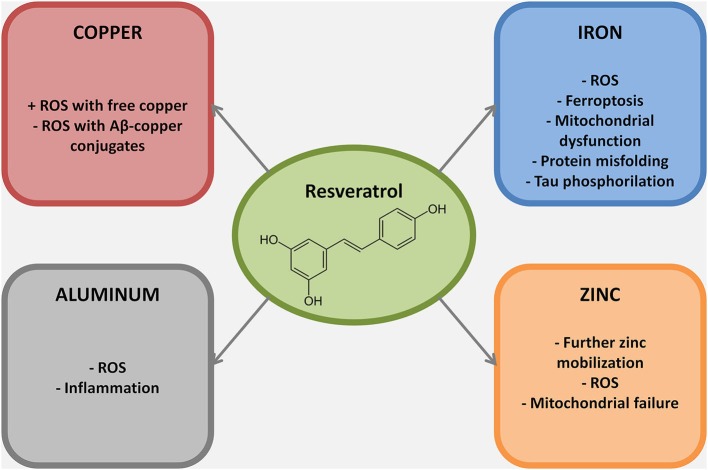
**Metal ions dyshomeostasis is closely related to different hallmarks of AD pathogenesis, mainly protein misfolding, ROS production, mitochondrial failure and inflammation**. In this figure the mechanisms through which resveratrol exert its neuroprotective role against selected metal ions are reported. Of note, most of the resveratrol beneficial effects against metal ion dyshomeostasis belong to its scavenging properties.

In summary, major effects are associated with its scavenging activity as well as in the activation of SIRT1 (see Bordone and Guarente, 2005; Herskovits and Guarente, 2014 for extensive reviews on the topic). The presence of non-SIRT1 neuronal targets of resveratrol is debated, suggesting that resveratrol *in vivo* may act on other uninvestigated biological targets (Herskovits and Guarente, 2014). The complementary role of modulator of metal dependent oxidative injury (Figure 2) represents a still largely unexplored field in resveratrol biochemistry.

Resveratrol is a multi-target, simple, safe, and cost-effective dietary supplement. Nevertheless, it should be reminded that its role as therapeutic agent is not devoid of potential problems. The pro-oxidant activity in presence of labile copper, the poor bioavailability and ease degradation all represent major issue that require new sophisticated efforts (Goldberg et al., 2003). Synthesis of novel resveratrol analogs is ongoing and improvement of drug delivery might represent in that regard the major targets to be considered in order to overcome current resveratrol limitations. In agreement, pterostilbene, a resveratrol derivative, has shown promise in preclinical models of neurodegeneration, resulting more efficient than resveratrol itself in modifying AD- and aging-related cognitive decline (Joseph et al., 2008; Chang et al., 2012). These results leave the door open for the use of newly synthesized resveratrol analogs in aging-related disorders (Bourzac, 2012; Ogas et al., 2013; Pezzuto et al., 2013).

## Conflict of interest statement

The authors declare that the research was conducted in the absence of any commercial or financial relationships that could be construed as a potential conflict of interest.

## References

[B1] Ayton S. Lei P. Bush A. I. (2013). Metallostasis in Alzheimer’s disease. Free Radic. Biol. Med. 62, 76–89 10.1016/j.freeradbiomed.2012.10.558 23142767

[B2] Baur J. A. Sinclair D. A. (2006). Therapeutic potential of resveratrol: the in vivo evidence. Nat. Rev. Drug Discov. 5, 493–506 10.1038/nrd2060 16732220

[B3] Belguendouz L. Fremont L. Linard A. (1997). Resveratrol inhibits metal ion-dependent and independent peroxidation of porcine low-density lipoproteins. Biochem. Pharmacol. 53, 1347–1355 10.1016/s0006-2952(96)00820-9 9214696

[B4] Billard J. M. (2006). Ageing, hippocampal synaptic activity and magnesium. Magnes. Res. 19, 199–215 10.1684/mrh.2006.0063 17172010

[B5] Boillee S. Vande Velde C. Cleveland D. W. (2006). ALS: a disease of motor neurons and their nonneuronal neighbors. Neuron 52, 39–59 10.1016/j.neuron.2006.09.018 17015226

[B6] Bolognin S. Messori L. Zatta P. (2009). Metal ion physiopathology in neurodegenerative disorders. Neuromolecular Med. 11, 223–238 10.1007/s12017-009-8102-1 19946766

[B7] Bolognin S. Messori L. Drago D. Gabbiani C. Cendron L. Zatta P. (2011). Aluminum, copper, iron and zinc differentially alter amyloid-Abeta (1–42) aggregation and toxicity. Int. J. Biochem. Cell Biol. 43, 877–885 10.1016/j.biocel.2011.02.009 21376832

[B8] Bolognin S. Zatta P. Lorenzetto E. Valenti M. T. Buffelli M. (2013). β-Amyloid-aluminum complex alters cytoskeletal stability and increases ROS production in cortical neurons. Neurochem. Int. 62, 566–574 10.1016/j.neuint.2013.02.008 23416043

[B9] Bordone L. Guarente L. (2005). Calorie restriction, SIRT1 and metabolism: understanding longevity. Nat. Rev. Mol. Cell Biol. 6, 298–305 10.1038/nrm1616 15768047

[B10] Bourzac K. (2012). Interventions: live long and prosper. Nature 492, S18–S20 10.1038/492s18a 23222670

[B11] Brewer G. J. (2012). Copper excess, zinc deficiency and cognition loss in Alzheimer’s disease. Biofactors 38, 107–113 10.1002/biof.1005 22438177

[B12] Breydo L. Uversky V. N. (2011). Role of metal ions in aggregation of intrinsically disordered proteins in neurodegenerative diseases. Metallomics 3, 1163–1180 10.1039/c1mt00106j 21869995

[B13] Bush A. I. (2008). Drug development based on the metals hypothesis of Alzheimer’s disease. J. Alzheimers Dis. 15, 223–240 18953111 10.3233/jad-2008-15208

[B14] Cai Z. Yan L. J. Ratka A. (2013). Telomere shortening and Alzheimer’s disease. Neuromolecular Med. 15, 25–48 10.1007/s12017-012-8207-9 23161153

[B15] Capiralla H. Vingtdeux V. Zhao H. Sankowski R. Al-Abed Y. Davies P. (2012). Resveratrol mitigates lipopolysaccharide- and Abeta-mediated microglial inflammation by inhibiting the TLR4/NF-kappaB/STAT signaling cascade. J. Neurochem. 120, 461–472 10.1111/j.1471-4159.2011.07594.x 22118570 PMC3253186

[B16] Chang J. Rimando A. Pallas M. Camins A. Porquet D. Reeves J. (2012). Low-dose pterostilbene, but not resveratrol, is a potent neuromodulator in aging and Alzheimer’s disease. Neurobiol. Aging 33, 2062–2071 10.1016/j.neurobiolaging.2011.08.015 21982274

[B17] Chen M. L. Yi L. Jin X. Liang X. Y. Zhou Y. Zhang T. (2013). Resveratrol attenuates vascular endothelial inflammation by inducing autophagy through the cAMP signaling pathway. Autophagy 9, 2033–2045 10.4161/auto.26336 24145604

[B18] Chen W. T. Liao Y. H. Yu H. M. Cheng I. H. Chen Y. R. (2011). Distinct effects of Zn2+, Cu2+, Fe3+ and Al3+ on amyloid-beta stability, oligomerization and aggregation: amyloid-beta destabilization promotes annular protofibril formation. J. Biol. Chem. 286, 9646–9656 10.1074/jbc.m110.177246 21216965 PMC3059000

[B19] Choi K. M. Lee H. L. Kwon Y. Y. Kang M. S. Lee S. K. Lee C. K. (2013). Enhancement of mitochondrial function correlates with the extension of lifespan by caloric restriction and caloric restriction mimetics in yeast. Biochem. Biophys. Res. Commun. 441, 236–242 10.1016/j.bbrc.2013.10.049 24141116

[B20] ClinicalTrials.gov [Internet]. (2014). Bethesda (MD): National Library of Medicine (US) [Accessed].

[B21] Corona G. Vauzour D. Hercelin J. Williams C. M. Spencer J. P. E. (2013). Phenolic acid intake, delivered via moderate champagne wine consumption, improves spatial working memory via the modulation of hippocampal and cortical protein expression/activation. Antioxid. Redox Signal. 19, 1676–1689 10.1089/ars.2012.5142 23458470

[B22] Crespo A. C. Silva B. Marques L. Marcelino E. Maruta C. Costa S. (2014). Genetic and biochemical markers in patients with Alzheimer’s disease support a concerted systemic iron homeostasis dysregulation. Neurobiol. Aging 35, 777–785 10.1016/j.neurobiolaging.2013.10.078 24199959

[B23] Crouch P. J. White A. R. Bush A. I. (2007). The modulation of metal bio-availability as a therapeutic strategy for the treatment of Alzheimer’s disease. FEBS J. 274, 3775–3783 10.1111/j.1742-4658.2007.05918.x 17617225

[B24] de la Lastra C. A. Villegas I. (2007). Resveratrol as an antioxidant and pro-oxidant agent: mechanisms and clinical implications. Biochem. Soc. Trans. 35, 1156–1160 10.1042/bst0351156 17956300

[B25] Desquiret-Dumas V. Gueguen N. Leman G. Baron S. Nivet-Antoine V. Chupin S. (2013). Resveratrol induces a mitochondrial complex I-dependent increase in NADH oxidation responsible for sirtuin activation in liver cells. J. Biol. Chem. 288, 36662–36675 10.1074/jbc.M113.466490 24178296 PMC3868777

[B26] Dixon S. J. Lemberg K. M. Lamprecht M. R. Skouta R. Zaitsev E. M. Gleason C. E. (2012). Ferroptosis: an iron-dependent form of nonapoptotic cell death. Cell 149, 1060–1072 10.1016/j.cell.2012.03.042 22632970 PMC3367386

[B27] Donmez G. (2012). The neurobiology of sirtuins and their role in neurodegeneration. Trends Pharmacol. Sci. 33, 494–501 10.1016/j.tips.2012.05.007 22749331

[B28] Donmez G. Wang D. Cohen D. E. Guarente L. (2010). SIRT1 suppresses beta-amyloid production by activating the alpha-secretase gene ADAM10. Cell 142, 320–332 10.1016/j.cell.2010.06.020 20655472 PMC2911635

[B29] Doody R. S. Thomas R. G. Farlow M. Iwatsubo T. Vellas B. Joffe S. (2014). Phase 3 trials of solanezumab for mild-to-moderate Alzheimer’s disease. N. Engl. J. Med. 370, 311–321 10.1056/NEJMoa1312889 24450890

[B30] Duce J. A. Bush A. I. (2010). Biological metals and Alzheimer’s disease: implications for therapeutics and diagnostics. Prog. Neurobiol. 92, 1–18 10.1016/j.pneurobio.2010.04.003 20444428

[B31] Duce J. A. Tsatsanis A. Cater M. A. James S. A. Robb E. Wikhe K. (2010). Iron-export ferroxidase activity of beta-amyloid precursor protein is inhibited by zinc in Alzheimer’s disease. Cell 142, 857–867 10.1016/j.cell.2010.08.014 20817278 PMC2943017

[B32] Faux N. G. Ritchie C. W. Gunn A. Rembach A. Tsatsanis A. Bedo J. (2010). PBT2 rapidly improves cognition in Alzheimer’s disease: additional phase II analyses. J. Alzheimers Dis. 20, 509–516 10.3233/JAD-2010-1390 20164561

[B33] Finney L. A. O’halloran T. V. (2003). Transition metal speciation in the cell: insights from the chemistry of metal ion receptors. Science 300, 931–936 10.1126/science.1085049 12738850

[B34] Frozza R. L. Bernardi A. Hoppe J. B. Meneghetti A. B. Battastini A. M. Pohlmann A. R. (2013). Lipid-core nanocapsules improve the effects of resveratrol against Abeta-induced neuroinflammation. J. Biomed. Nanotechnol. 9, 2086–2104 10.1166/jbn.2013.1709 24266263

[B35] Gaier E. D. Eipper B. A. Mains R. E. (2013). Copper signaling in the mammalian nervous system: synaptic effects. J. Neurosci. Res. 91, 2–19 10.1002/jnr.23143 23115049 PMC3926505

[B36] Giuliani D. Bitto A. Galantucci M. Zaffe D. Ottani A. Irrera N. (2014). Melanocortins protect against progression of Alzheimer’s disease in triple-transgenic mice by targeting multiple pathophysiological pathways. Neurobiol. Aging 35, 537–547 10.1016/j.neurobiolaging.2013.08.030 24094579

[B37] Goldberg D. M. Yan J. Soleas G. J. (2003). Absorption of three wine-related polyphenols in three different matrices by healthy subjects. Clin. Biochem. 36, 79–87 10.1016/s0009-9120(02)00397-1 12554065

[B38] Gomes A. P. Price N. L. Ling A. J. Moslehi J. J. Montgomery M. K. Rajman L. (2013). Declining NAD(+) induces a pseudohypoxic state disrupting nuclear-mitochondrial communication during aging. Cell 155, 1624–1638 10.1016/j.cell.2013.11.037 24360282 PMC4076149

[B39] Gonzalez-Dominguez R. Garcia-Barrera T. Gomez-Ariza J. L. (2014). Characterization of metal profiles in serum during the progression of Alzheimer’s disease. Metallomics 6, 292–300 10.1039/c3mt00301a 24343096

[B40] Granzotto A. Bolognin S. Scancar J. Milacic R. Zatta P. (2011). β-amyloid toxicity increases with hydrophobicity in the presence of metal ions. Monatsh. Chem. 142, 421–430 10.1007/s00706-011-0470-1

[B41] Granzotto A. Zatta P. (2011). Resveratrol acts not through anti-aggregative pathways but mainly via its scavenging properties against Abeta and Abeta-metal complexes toxicity. PLoS One 6:e21565 10.1371/journal.pone.0021565 21738712 PMC3124535

[B42] Halagappa V. K. Guo Z. Pearson M. Matsuoka Y. Cutler R. G. Laferla F. M. (2007). Intermittent fasting and caloric restriction ameliorate age-related behavioral deficits in the triple-transgenic mouse model of Alzheimer’s disease. Neurobiol. Dis. 26, 212–220 10.1016/j.nbd.2006.12.019 17306982

[B43] Hardy J. A. Higgins G. A. (1992). Alzheimer’s disease: the amyloid cascade hypothesis. Science 256, 184–185 10.1126/science.1566067 1566067

[B44] Heneka M. T. Kummer M. P. Stutz A. Delekate A. Schwartz S. Vieira-Saecker A. (2013). NLRP3 is activated in Alzheimer’s disease and contributes to pathology in APP/PS1 mice. Nature 493, 674–678 10.1038/nature11729 23254930 PMC3812809

[B45] Herrup K. (2010). Reimagining Alzheimer’s disease—an age-based hypothesis. J. Neurosci. 30, 16755–16762 10.1523/JNEUROSCI.4521-10.2010 21159946 PMC3004746

[B46] Herskovits A. Z. Guarente L. (2014). SIRT1 in neurodevelopment and brain senescence. Neuron 81, 471–483 10.1016/j.neuron.2014.01.028 24507186 PMC4040287

[B47] Howitz K. T. Bitterman K. J. Cohen H. Y. Lamming D. W. Lavu S. Wood J. G. (2003). Small molecule activators of sirtuins extend Saccharomyces cerevisiae lifespan. Nature 425, 191–196 10.1038/nature01960 12939617

[B48] James S. A. Volitakis I. Adlard P. A. Duce J. A. Masters C. L. Cherny R. A. (2012). Elevated labile Cu is associated with oxidative pathology in Alzheimer disease. Free Radic. Biol. Med. 52, 298–302 10.1016/j.freeradbiomed.2011.10.446 22080049

[B49] Jayasena T. Poljak A. Smythe G. Braidy N. Munch G. Sachdev P. (2013). The role of polyphenols in the modulation of sirtuins and other pathways involved in Alzheimer’s disease. Ageing Res. Rev. 12, 867–883 10.1016/j.arr.2013.06.003 23831960

[B50] Jellinger K. A. (2013). The relevance of metals in the pathophysiology of neurodegeneration, pathological considerations. Int. Rev. Neurobiol. 110, 1–47 10.1016/b978-0-12-410502-7.00002-8 24209432

[B51] Joseph J. A. Fisher D. R. Cheng V. Rimando A. M. Shukitt-Hale B. (2008). Cellular and behavioral effects of stilbene resveratrol analogues: implications for reducing the deleterious effects of aging. J. Agric. Food Chem. 56, 10544–10551 10.1021/jf802279h 18954071

[B52] Khan R. S. Fonseca-Kelly Z. Callinan C. Zuo L. Sachdeva M. M. Shindler K. S. (2012). SIRT1 activating compounds reduce oxidative stress and prevent cell death in neuronal cells. Front. Cell. Neurosci. 6:63 10.3389/fncel.2012.00063 23293585 PMC3533205

[B53] Kim D. Nguyen M. D. Dobbin M. M. Fischer A. Sananbenesi F. Rodgers J. T. (2007). SIRT1 deacetylase protects against neurodegeneration in models for Alzheimer’s disease and amyotrophic lateral sclerosis. EMBO J. 26, 3169–3179 10.1038/sj.emboj.7601758 17581637 PMC1914106

[B54] Krstic D. Knuesel I. (2013). Deciphering the mechanism underlying late-onset alzheimer disease. Nat. Rev. Neurol. 9, 25–34 10.1038/nrneurol.2012.236 23183882

[B55] Kumar V. Bal A. Gill K. D. (2009). Susceptibility of mitochondrial superoxide dismutase to aluminium induced oxidative damage. Toxicology 255, 117–123 10.1016/j.tox.2008.10.009 19010380

[B56] Lagouge M. Argmann C. Gerhart-Hines Z. Meziane H. Lerin C. Daussin F. (2006). Resveratrol improves mitochondrial function and protects against metabolic disease by activating SIRT1 and PGC-1alpha. Cell 127, 1109–1122 10.1016/j.cell.2006.11.013 17112576

[B57] Leonard S. S. Xia C. Jiang B. H. Stinefelt B. Klandorf H. Harris G. K. (2003). Resveratrol scavenges reactive oxygen species and effects radical-induced cellular responses. Biochem. Biophys. Res. Commun. 309, 1017–1026 10.1016/j.bbrc.2003.08.105 13679076

[B58] Liu T. Bitan G. (2012). Modulating self-assembly of amyloidogenic proteins as a therapeutic approach for neurodegenerative diseases: strategies and mechanisms. ChemMedChem 7, 359–374 10.1002/cmdc.201100585 22323134

[B59] Liu G. Men P. Zhu X. Perry G. (2012). Iron chelation and nanoparticle target delivery in the development of new multifunctional disease-modifying drugs for Alzheimer’s disease. Ther. Deliv. 3, 571–574 10.4155/tde.12.32 22834400

[B60] López-Otín C. Blasco M. A. Partridge L. Serrano M. Kroemer G. (2013). The hallmarks of aging. Cell 153, 1194–1217 10.1016/j.cell.2013.05.039 23746838 PMC3836174

[B61] Marambaud P. Zhao H. Davies P. (2005). Resveratrol promotes clearance of Alzheimer’s disease amyloid-beta peptides. J. Biol. Chem. 280, 37377–37382 10.1074/jbc.m508246200 16162502

[B62] Mathur S. Glogowska A. Mcavoy E. Righolt C. Rutherford J. Willing C. (2014). Three-dimensional quantitative imaging of telomeres in buccal cells identifies mild, moderate and severe Alzheimer’s disease patients. J. Alzheimers Dis. 39, 35–48 10.3233/JAD-130866 24121960

[B63] McGeer P. L. McGeer E. G. (2013). The amyloid cascade-inflammatory hypothesis of Alzheimer disease: implications for therapy. Acta Neuropathol. 126, 479–497 10.1007/s00401-013-1177-7 24052108

[B64] Medeiros R. Chabrier M. A. Laferla F. M. (2013). Elucidating the triggers, progression and effects of Alzheimer’s disease. J. Alzheimers Dis. 33(Suppl. 1), S195–S210 10.3233/JAD-2012-129009 22635105

[B65] Min S. W. Cho S. H. Zhou Y. Schroeder S. Haroutunian V. Seeley W. W. (2010). Acetylation of tau inhibits its degradation and contributes to tauopathy. Neuron 67, 953–966 10.1016/j.neuron.2010.08.044 20869593 PMC3035103

[B66] Mucke L. Selkoe D. J. (2012). Neurotoxicity of amyloid beta-protein: synaptic and network dysfunction. Cold Spring Harb. Perspect. Med. 2:a006338 10.1101/cshperspect.a006338 22762015 PMC3385944

[B67] Mudher A. Lovestone S. (2002). Alzheimer’s disease-do tauists and baptists finally shake hands? Trends Neurosci. 25, 22–26 10.1016/s0166-2236(00)02031-2 11801334

[B68] Muqbil I. Beck F. W. Bao B. Sarkar F. H. Mohammad R. M. Hadi S. M. (2012). Old wine in a new bottle: the Warburg effect and anticancer mechanisms of resveratrol. Curr. Pharm. Des. 18, 1645–1654 10.2174/138161212799958567 22288443

[B69] Ogas T. Kondratyuk T. P. Pezzuto J. M. (2013). Resveratrol analogs: promising chemopreventive agents. Ann. N Y Acad. Sci. 1290, 21–29 10.1111/nyas.12196 23855462

[B106] Palacios J. A. Herranz D. De Bonis M. L. Velasco S. Serrano M. Blasco M. A. (2010). SIRT1 contributes to telomere maintenance and augments global homologous recombination. J. Cell. Biol. 191, 1299–1313 10.1083/jcb.201005160 21187328 PMC3010065

[B70] Panza F. Frisardi V. Seripa D. Logroscino G. Santamato A. Imbimbo B. P. (2012). Alcohol consumption in mild cognitive impairment and dementia: harmful or neuroprotective? Int. J. Geriatr. Psychiatry 27, 1218–1238 10.1002/gps.3772 22396249

[B71] Park S. J. Ahmad F. Philp A. Baar K. Williams T. Luo H. (2012). Resveratrol ameliorates aging-related metabolic phenotypes by inhibiting cAMP phosphodiesterases. Cell 148, 421–433 10.1016/j.cell.2012.01.017 22304913 PMC3431801

[B72] Perl D. P. (2006). Exposure to aluminium and the subsequent development of a disorder with features of Alzheimer’s disease. J. Neurol. Neurosurg. Psychiatry 77:811 10.1136/jnnp.2006.090613 16627536 PMC2117484

[B73] Pezzuto J. M. Kondratyuk T. P. Ogas T. (2013). Resveratrol derivatives: a patent review (2009 - 2012). Expert Opin. Ther. Pat. 23, 1529–1546 10.1517/13543776.2013.834888 24032623

[B74] Pithadia A. S. Lim M. H. (2012). Metal-associated amyloid-beta species in Alzheimer’s disease. Curr. Opin. Chem. Biol. 16, 67–73 10.1016/j.cbpa.2012.01.016 22366383

[B75] Porat Y. Abramowitz A. Gazit E. (2006). Inhibition of amyloid fibril formation by polyphenols: structural similarity and aromatic interactions as a common inhibition mechanism. Chem. Biol. Drug Des. 67, 27–37 10.1111/j.1747-0285.2005.00318.x 16492146

[B76] Querfurth H. W. Laferla F. M. (2010). Alzheimer’s disease. N. Engl. J. Med. 362, 329–344 10.1056/NEJMra0909142 20107219

[B77] Rahman I. Biswas S. K. Kirkham P. A. (2006). Regulation of inflammation and redox signaling by dietary polyphenols. Biochem. Pharmacol. 72, 1439–1452 10.1016/j.bcp.2006.07.004 16920072

[B78] Reitz C. (2012). Alzheimer’s disease and the amyloid cascade hypothesis: a critical review. Int. J. Alzheimers Dis. 2012:369808 10.1155/2012/369808 22506132 PMC3313573

[B79] Renaud S. De Lorgeril M. (1992). Wine, alcohol, platelets and the French paradox for coronary heart disease. Lancet 339, 1523–1526 10.1016/0140-6736(92)91277-f 1351198

[B80] Rivière C. Richard T. Quentin L. Krisa S. Merillon J. M. Monti J. P. (2007). Inhibitory activity of stilbenes on Alzheimer’s beta-amyloid fibrils in vitro. Bioorg. Med. Chem. 15, 1160–1167 10.1016/j.bmc.2006.09.069 17049256

[B81] Rizzuto R. De Stefani D. Raffaello A. Mammucari C. (2012). Mitochondria as sensors and regulators of calcium signalling. Nat. Rev. Mol. Cell Biol. 13, 566–578 10.1038/nrm3412 22850819

[B82] Roberts B. R. Ryan T. M. Bush A. I. Masters C. L. Duce J. A. (2012). The role of metallobiology and amyloid-beta peptides in Alzheimer’s disease. J. Neurochem. 120(Suppl. 1), 149–166 10.1111/j.1471-4159.2011.07500.x 22121980

[B83] Sayre L. M. Perry G. Harris P. L. Liu Y. Schubert K. A. Smith M. A. (2000). In situ oxidative catalysis by neurofibrillary tangles and senile plaques in Alzheimer’s disease: a central role for bound transition metals. J. Neurochem. 74, 270–279 10.1046/j.1471-4159.2000.0740270.x 10617129

[B84] Sekler I. Silverman W. F. (2012). Zinc homeostasis and signaling in glia. Glia 60, 843–850 10.1002/glia.22286 22328236

[B85] Sensi S. L. Paoletti P. Bush A. I. Sekler I. (2009). Zinc in the physiology and pathology of the CNS. Nat. Rev. Neurosci. 10, 780–791 10.1038/nrn2734 19826435

[B86] Sharma D. R. Sunkaria A. Wani W. Y. Sharma R. K. Kandimalla R. J. Bal A. (2013). Aluminium induced oxidative stress results in decreased mitochondrial biogenesis via modulation of PGC-1alpha expression. Toxicol. Appl. Pharmacol. 273, 365–380 10.1016/j.taap.2013.09.012 24084166

[B87] Shin S. M. Cho I. J. Kim S. G. (2009). Resveratrol protects mitochondria against oxidative stress through AMP-activated protein kinase-mediated glycogen synthase kinase-3beta inhibition downstream of poly(ADP-ribose)polymerase-LKB1 pathway. Mol. Pharmacol. 76, 884–895 10.1124/mol.109.058479 19620254

[B88] Siemann E. H. Creasy L. L. (1992). Concentration of the Phytoalexin resveratrol in wine. Am. J. Enol. Vitic. 43, 49–52

[B89] Singh I. Sagare A. P. Coma M. Perlmutter D. Gelein R. Bell R. D. (2013). Low levels of copper disrupt brain amyloid-beta homeostasis by altering its production and clearance. Proc. Natl. Acad. Sci. U S A 110, 14771–14776 10.1073/pnas.1302212110 23959870 PMC3767519

[B90] Tadolini B. Juliano C. Piu L. Franconi F. Cabrini L. (2000). Resveratrol inhibition of lipid peroxidation. Free Radic. Res. 33, 105–114 10.1080/10715760000300661 10826926

[B91] Tamboli V. Defant A. Mancini I. Tosi P. (2011). A study of resveratrol-copper complexes by electrospray ionization mass spectrometry and density functional theory calculations. Rapid Commun. Mass Spectrom. 25, 526–532 10.1002/rcm.4883 21259361

[B92] Uchiumi F. Watanabe T. Hasegawa S. Hoshi T. Higami Y. Tanuma S. (2011). The effect of resveratrol on the Werner syndrome RecQ helicase gene and telomerase activity. Curr. Aging Sci. 4, 1–7 10.2174/1874612811104010001 21204775

[B93] Vingtdeux V. Giliberto L. Zhao H. Chandakkar P. Wu Q. Simon J. E. (2010). AMP-activated protein kinase signaling activation by resveratrol modulates amyloid-beta peptide metabolism. J. Biol. Chem. 285, 9100–9113 10.1074/jbc.m109.060061 20080969 PMC2838330

[B94] Walton J. R. (2013). Aluminum involvement in the progression of Alzheimer’s disease. J. Alzheimers Dis. 35, 7–43 10.3233/JAD-121909 23380995

[B95] Wang J. Ho L. Zhao Z. Seror I. Humala N. Dickstein D. L. (2006). Moderate consumption of Cabernet Sauvignon attenuates A beta neuropathology in a mouse model of Alzheimer’s disease. FASEB J. 20, 2313–2320 10.1096/fj.06-6281com 17077308

[B96] Wang X. Wang W. Li L. Perry G. Lee H. G. Zhu X. (2013). Oxidative stress and mitochondrial dysfunction in Alzheimer’s disease. Biochim. Biophys. Acta [Epub ahead of print]. 10.1016/j.bbadis.2013.10.015 24189435 PMC4007397

[B97] Weinreb O. Mandel S. Youdim M. B. Amit T. (2013). Targeting dysregulation of brain iron homeostasis in Parkinson’s disease by iron chelators. Free Radic. Biol. Med. 62, 52–64 10.1016/j.freeradbiomed.2013.01.017 23376471

[B98] Weinstein G. Beiser A. S. Choi S. H. Preis S. R. Chen T. C. Vorgas D. (2014). Serum brain-derived neurotrophic factor and the risk for dementia: the Framingham Heart Study. JAMA Neurol. 71, 55–61 10.1001/jamaneurol.2013.4781 24276217 PMC4056186

[B99] Wu Z. Du Y. Xue H. Wu Y. Zhou B. (2012). Aluminum induces neurodegeneration and its toxicity arises from increased iron accumulation and reactive oxygen species (ROS) production. Neurobiol. Aging 33, 199.e1–199.e12 10.1016/j.neurobiolaging.2010.06.018 20674094

[B100] Ye J. Liu Z. Wei J. Lu L. Huang Y. Luo L. (2013). Protective effect of SIRT1 on toxicity of microglial-derived factors induced by LPS to PC12 cells via the p53-caspase-3-dependent apoptotic pathway. Neurosci. Lett. 553, 72–77 10.1016/j.neulet.2013.08.020 23973301

[B101] Zaky A. Mohammad B. Moftah M. Kandeel K. M. Bassiouny A. R. (2013). Apurinic/apyrimidinic endonuclease 1 is a key modulator of aluminum-induced neuroinflammation. BMC Neurosci. 14:26 10.1186/1471-2202-14-26 23497276 PMC3616857

[B102] Zatta P. Drago D. Bolognin S. Sensi S. L. (2009). Alzheimer’s disease, metal ions and metal homeostatic therapy. Trends Pharmacol. Sci. 30, 346–355 10.1016/j.tips.2009.05.002 19540003

[B103] Zatta P. Kiss T. Suwalsky M. Berthon G. (2002). Aluminium(III) as a promoter of cellular oxidation. Coord. Chem. Rev. 228, 271–284 10.1016/S0010-8545(02)00074-7

[B104] Zatta P. Lucchini R. Van Rensburg S. J. Taylor A. (2003). The role of metals in neurodegenerative processes: aluminum, manganese, and zinc. Brain Res. Bull. 62, 15–28 10.1016/s0361-9230(03)00182-5 14596888

[B105] Zheng L. F. Wei Q. Y. Cai Y. J. Fang J. G. Zhou B. Yang L. (2006). DNA damage induced by resveratrol and its synthetic analogues in the presence of Cu (II) ions: mechanism and structure-activity relationship. Free Radic. Biol. Med. 41, 1807–1816 10.1016/j.freeradbiomed.2006.09.007 17157183

